# Mental Health Benefits of Long-Term Exposure to Residential Green and Blue Spaces: A Systematic Review

**DOI:** 10.3390/ijerph120404354

**Published:** 2015-04-22

**Authors:** Mireia Gascon, Margarita Triguero-Mas, David Martínez, Payam Dadvand, Joan Forns, Antoni Plasència, Mark J. Nieuwenhuijsen

**Affiliations:** 1 ISGlobal, Barcelona Ctr. Int. Health Res. (CRESIB), Hospital Clínic-Universitat de Barcelona, Barcelona 08036, Spain; E-Mail: antoni.plasencia@isglobal.org; 2 Parc de Recerca Biomèdica de Barcelona (PRBB), Centre for Research in Environmental Epidemiology (CREAL). Doctor Aiguader, 88, 08003 Barcelona, Catalonia, Spain; E-Mails: mtriguero@creal.cat (M.T.-M.); dmartinez@creal.cat (D.M.); pdadvand@creal.cat (P.D.); jforns@creal.cat (J.F.); mnieuwenhuijsen@creal.cat (M.J.N.); 3 CIBER Epidemiología y Salud Pública (CIBERESP), Barcelona 08036, Spain; 4 Department of Genes and Environment, Division of Epidemiology, Norwegian Institute of Public Health, Oslo 0403, Norway

**Keywords:** green spaces, blue spaces, mental health

## Abstract

Many studies conducted during the last decade suggest the mental health benefits of green and blue spaces. We aimed to systematically review the available literature on the long-term mental health benefits of residential green and blue spaces by including studies that used standardized tools or objective measures of both the exposures and the outcomes of interest. We followed the PRISMA statement guidelines for reporting systematic reviews and meta-analysis. In total 28 studies were included in the systematic review. We found limited evidence for a causal relationship between surrounding greenness and mental health in adults, whereas the evidence was inadequate in children. The evidence was also inadequate for the other exposures evaluated (access to green spaces, quality of green spaces, and blue spaces) in both adults and children. The main limitation was the limited number of studies, together with the heterogeneity regarding exposure assessment. Given the increase in mental health problems and the current rapid urbanization worldwide, results of the present systematic review should be taken into account in future urban planning. However, further research is needed to provide more consistent evidence and more detailed information on the mechanisms and the characteristics of the green and blue spaces that promote better mental health. We provide recommendations for future studies in order to provide consistent and evidence-based recommendations for policy makers.

## 1. Introduction

Mental, neurological and substance use disorders account for 13% of the total global burden of disease [1]. A recent study reported that the global cost of mental health disorders in 2010 was an estimated US$ 2.5 trillion, and that these costs can raise to US$ 6.0 trillion by 2030 [2]. Many factors play a role in the occurrence of mental health disorders, including multiple social, psychological, and biological factors, as well as the environment in where we live, particularly the characteristics of the urban environment [1]. About half of the world population is currently living in cities and it is projected that by 2030 three of every five persons will live in urban areas worldwide [3]. Living in a city can be beneficial for people’s well-being as it facilitates the establishment of social networks and access to several services, including health care services. However, some aspects of living in a city can be detrimental for people's health (e.g., air pollution, space restrictions, noise), and these city detrimental aspects may increase in the coming years [4].

Green and blue spaces within cities have been associated with better mental health conditions (e.g., less risk of depression symptoms, psychological distress, *etc.*) [5,6,7]. The term green spaces refers to vegetation (trees, grass, forests, parks, *etc.*), whereas blue spaces are all the visible surface waters in space (lakes, rivers, coastal water). The European Commission recommends that open public spaces should be within a distance of 300 m of residences [8]. However, it is not clear yet which distance to green/blue spaces or what amount of surrounding greenness/blueness is actually relevant for a better mental health, nor is the weight that each determinant (access to green/blue spaces or surrounding greenness/blueness) has on the association between green/blue spaces and a better mental health condition. Indeed, the influence of the quality of these green/blue spaces on this association has been poorly explored. One of the main limitations to elucidate these questions is that many of the studies included in previous reviews used non-objective or non-standard tools to assess both residential green/blue spaces and mental health condition [5,9,10]. Additionally, many studies available are experimental and evaluate the short-term health effects of exposure to green/blue spaces [5,11,12] but not the health effects of long-term exposure to residential “greenness/blueness”, which is an essential information for policy makers to take appropriate decisions in urban planning. In the present study we aim to systematically review the long-term mental health benefits of residential green and blue spaces by including studies that use standardized tools or objective measures of both the exposures and the outcomes of interest.

## 2. Experimental Section

### 2.1. Search Strategy and Selection Criteria

We followed the Preferred Reporting Items for Systematic reviews and Meta-Analyses (PRISMA) statement guidelines for reporting systematic reviews and meta-analysis, a protocol that aims to help authors improve the reporting of systematic reviews and meta-analyses [13]. The bibliographic search was carried out by two independent reviewers (MG and MTM) through two of the most used search engines, MEDLINE (National Library of Medicine) and Scopus (Web of Science), using the following keywords, which were chosen based on the terms mostly used in this field of research: keywords related to *green and blue spaces* (greenspace, green space, natural environment, urban design, built environment, blue space, park, forest) combined with the following keywords related to *mental health* (mood disorder, dysthymic disorder, depressive disorder, depression, bipolar disorder, cyclothymic disorder, anxiety disorder, anxiety, panic disorder, agoraphobia, phobia, obsessive-compulsive disorder, posttraumatic stress, stress, acute stress disorder, somatisation disorder, somatoform disorder, hypochondriasis, body dysmorphic disorder, factitious disorder, depersonalization disorder, dissociative amnesia, dissociative disorder, mental health, mental hygiene, mental disorders, emotional well-being, psychological well-being, social well-being, well-being). Limits: The search was limited to the English language and studies in Humans and the last search was conducted on 11 October 2014. Identification and first screening of the articles was performed using the information available in the title and the abstract. Doubts regarding the inclusion or exclusion of studies were resolved by discussion between the two independent researchers and with the help of a third researcher. After the first selection, both reviewers read through the articles to decide whether they were eligible or not. We also checked the references of the relevant articles to find other articles following the inclusion criteria. During the revision process an additional paper was identified and included [14].

### 2.2. Study Eligibility Criteria

Following the methodology used in a previous review on green spaces and obesity [15], the selection criteria were: (a) the article was an original research article; (b) the article used empirical data to report analysis of mental health outcomes in relation to green or blue space exposure; (c) the green or blue space measures were generated using objective methods, either by use of remote sensing data, land use/land cover maps, or an assessment by trained auditors using a consistent tool; (d) green or blue space exposure was assigned based on location of residence; (e) green or blue space exposure was included as a separate variable within the analysis and results were reported specifically for green or blue space, even if these were not the primary aim of the study; (f) experimental studies which looked at interactions with nature or simulated views of nature were not included; and (h) the article was written in English.

### 2.3. Evaluation of Evidence

We first evaluated the basic characteristics and quality of the methodology of the studies included in the systematic review by extracting the following data: author, year of publication, country, study design, study population, sample size, exposure assessment, outcome assessment, confounding factors, and other relevant information including information on potential biases (Table 1 and Table 2 and see Supplemental Material, Table S1). The two reviewers independently worked on data extraction and evaluation of the quality of the studies. Agreement was reached via consensus and classified the evidence. In order to facilitate the classification of the evidence for a causal relationship between the exposures and the outcomes of interest we evaluated the quality of the studies and obtained a quality score for each study (See Supplemental Material, Tables S2 and S3) based on an adapted version of the criteria used in a previous review [15]. Briefly, this quality score was based on 11 different items which could score from 0 to 2 each (See Supplemental Material, Table S2 for further information). For each study the total score was calculated by adding the scores on the 11 dimensions and expressing them as a percentage of the maximum score (=14 or 12 in the case of two studies). Afterwards, five categories were created to define the quality of each study: *excellent quality* (score ≥81%), *good quality* (between 61% and 80%), *fair quality* (between 41% and 60%), *poor quality* (between 21% and 40%) and *very poor quality* (≤20%) (See Supplemental Material, Table S3). We separately evaluated the evidence according to the age of the targeted study population: (1) exclusively children and (2) adults, which can include population from 15 years onwards, or population irrespective of age. We also separately evaluated the evidence according to the type of exposure assessed: (1) surrounding greenness—the amount of greenness—e.g., coming from trees, grass, or bushes-within a certain distance from the residence; (2) access to green spaces—the presence of a green space—e.g., parks, forests, or other natural areas-within a walkable distance from the residence; (3) quality of green spaces—e.g., aesthetics, biodiversity, walkability, feeling of safety, type of trees, performance of social activities; and (4) blue spaces (amount, access to and quality)—e.g., lakes, rivers, or coastal water. Finally, we classified the strength of the evidence based on an adapted version of the guidelines for level of evidence used by the International Agency for Research on Cancer that has been previously used in other studies from the same field as this review [16]. Evidence for causal relationships for each exposure-outcome was classified as: *sufficient*-if most of the studies, including good quality studies, report an association, but evidence is not yet conclusive enough to conclude that there is a causal relationship, *limited*-several good quality, independent, studies report an association, but evidence is not yet conclusive enough, *inadequate*-if associations are reported in one or more studies, but insufficient quality, insufficient number of studies, lack of consistency, and/or lack of statistical power preclude a conclusion regarding the presence or absence of a causal relationship, *evidence for lack of association*-several good quality studies are consistent in showing no causal relationship.

## 3. Results

**Figure 1:**
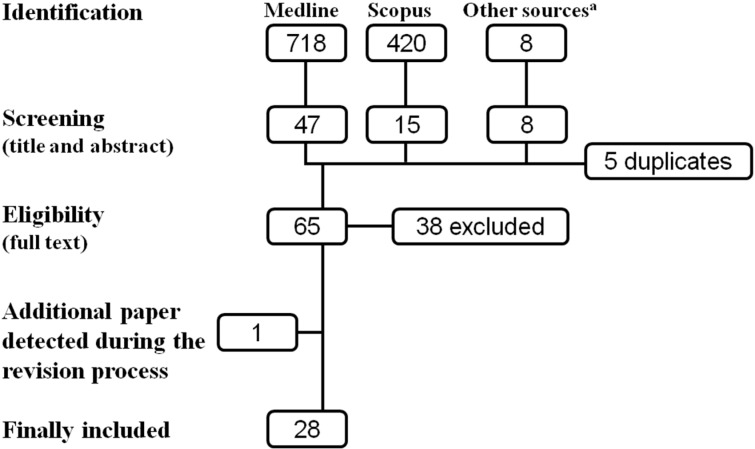
Selection process of the articles.

A total of 718 articles were identified in MEDLINE and 420 in Scopus. Through other sources eight articles were also identified. After screening the title and the abstracts and checking for duplicates, 65 articles were chosen for full-text evaluation, of which 27 articles were finally included in the systematic review. During the revision process an additional paper was identified and included [14]. Thus, in total 28 studies were finally included (Figure 1).

Six of the selected studies were longitudinal studies [17,18,19,20,21,22], one was an ecological study [23] and the rest were cross-sectional studies. Five studies were classified as good quality studies [18,20,22,24,25], but most of the studies were considered to be of fair quality and only two of poor quality [26,27] (See Supplemental material, Table S3). Nineteen of the 28 studies were conducted in Europe, mainly in the United Kingdom (*N* = 8) and The Netherlands (*N* = 5). The rest of the studies were conducted in The United States (*N* = 4) or Oceania (*N* = 4). Only one study was conducted in a Latin American country and none in Asia or Africa. The size of the study populations was very heterogeneous among studies, ranging from ~100 to 345,143 participants (See Supplemental material, Table S1).

Four studies included only children (from 3 to 10 years of age) and evaluated emotional and behavioural problems through the Strengths and Difficulties Questionnaire (SDQ) and/or the ADHD symptom Criteria of Diagnostic and Statistical Manual of Mental Health (ADHD/DSM-IV) [19,28,29,30] (Table 1 and Table 2). We did not find studies assessing cognitive or psychomotor development in children in relation to exposure to green or blue spaces.

Half of the studies including adults used the General Health Questionnaire (GHQ) [14,17,18,20,21,22,34,35,38,39], the Mental Health Inventory (MHI) [42] or the Short Form health survey (SF) [14,36] to evaluate general mental health. The other half focused on more specific disorders such as stress, distress, depression, anxiety and mood disorders [23,24,25,26,27,31,32,33,37,40,41,43], assessed with different tools (Table 1 and Table 2).

**Table 1:** Main characteristics and results of the studies on surrounding greenness and mental health.

Author (Year, Country)	Study Design	Age of the Study Population (Stratifications/Interactions)	N	Tools to Measure Mental Health	Mental Health Item	Greenness Data Source	Surrounding Greenness Indicator	Risk of Mental Health Problems
Exclusively children								
Amoly 2014 *et al.*, Spain [30]	Cross-sectional	Children 7–10 y	2111	SDQ ADHD/DSM-IV	Emotional & behavioural problems ^a^	NDVI	100 m, 250 m, 500 m buffers	Increasing greenness 100 m buffer: ↓ total SDQ difficulties, SDQ hyperactivity/inattention & ADHD (inattention) 250 m buffer: ↓ total SDQ difficulties, SDQ hyperactivity/inattention 500 m buffer: ↓ total SDQ difficulties, SDQ hyperactivity/inattention, SDQ emotional symptoms
Balseviciene *et al.* 2014, Lithuania [28]	Cross-sectional	4–6 y (maternal education)	1468	SDQ	Emotional & behavioural problems ^a^	NDVI	300 m buffer	Higher maternal education group: increasing greenness ↑ conditional problems & ↓ prosocial behaviour
Flouri *et al.* 2014, The UK [19]	Longitudinal	3, 5 & 7 y (socioeconomic status)	6384	SDQ	Emotional & behavioural problems ^a^	Land-cover map	% GS at CAU	Poor children of age 3y to 5y: increasing greenness ↓ emotional problems
Markevych *et al.* 2014, Germany [29]	Cross-sectional	10 y (gender, urbanity degree)	1932	SDQ	Emotional & behavioural problems ^a^	NDVI	500 m buffer	-
Adults (or population irrespective of age)								
Alcock *et al.* 2014, The UK [22]	Longitudinal	Adults	1064	GHQ-12	Mental health	Land-cover map	% GS at CAU (residence change in time)	↑ mental health in people moving to greener areas
Araya *et al.* 2007, Chile [31]	Cross-sectional	Adults 16–64 y	3870	CIS-R ICD-10	Psychiatric, anxiety and depressive disorders	BEAT (audit)	Presence of public green areas and its quality ^b^ at CAU	Increasing presence of public green areas ↓ risk of depression (ICD-10)
Astell-Burt *et al.* 2013, Australia [32]	Cross-sectional	>45 y (physical activity)	260061	K10	Psychological distress	Land-cover map	% GS in 1 km buffer	Increasing greenness ↓ risk in all population (after stratification only in physically active adults)
Astell-Burt *et al.* 2014, The UK [18]	Longitudinal	>15 y (age, gender)	65407	GHQ-12	Minor psychiatric morbidity	Land-cover map	% GS at CAU	Increasing greenness ↓ risk in males >30 years and in females >41 years & living in moderate greenness
Beyer *et al.* 2014, The USA [33]	Cross-sectional	21–74 y	2479	DASS	Depression Anxiety Stress	NDVI	At CAU	Increasing greenness ↓ risk of depression & anxiety
						Land-cover map	% tree canopy coverage at CAU	Increasing greenness ↓ risk of depression & stress
De Vries *et al.* 2003, The Netherlands [34]	Cross-sectional	All ages (education, urbanity degree)	10197	GHQ	Minor psychiatric morbidity	Land-cover map	% GS in 1 km & 3 km buffers	Increasing greenness between 1 and 3 km ↓ risk in all population (after stratification only in low educated)
Fan *et al.* 2011, The USA [27]	Cross-sectional	Adults 18–75 y	1544	PSS	Stress	NDVI	800 m buffer	-
						Land-cover map	Total park acreage in a 800 m buffer	-
Adults (or population irrespective of age)								
Francis *et al.* 2012, Australia [24]	Cross-sectional	Adults (20–79 y)	911	K6	Psychological distress	Land-cover map	Size and n° of public open space in a 1600 m buffer	-
Maas *et al.* 2009, The Netherlands [35]	Cross-sectional	12 to >65 y	4842-10089	GHQ-12	Propensity to psychiatric morbidity	Land-cover map	% GS in 1 km & 3 km buffers	Increasing greenness in 1 km ↓ propensity to psychiatric morbidity
Maas *et al.* 2009, The Netherlands [25]	Cross-sectional	All ages (age, socioeconomic status, urbanity degree)	345143	Primary care medical records	Mental health morbidity (depression and anxiety)	Land-cover map	% GS in 1 km & 3 km buffers	Increasing greenness in 1 km ↓ depression & anxiety In 3 km ↓ anxiety (stronger associations with depression in children for both buffers)
Nutsford *et al.* 2013, New Zealand [23]	Ecological	>15 y	319521	Health ministry database	Anxiety/mood disorder treatment counts	Land-cover map	% of total and useable GS of >500 m^2^ (300 m & 3 km buffers)	Increasing total and usable GS in 3 km ↓ risk of treatment
Richardson *et al.* 2013, New Zealand [36]	Cross-sectional	>15 y (physical activity)	8157	SF-36	Mental health	Land-cover map	% GS of ≥0.02 ha at CAU	Increasing greenness ↓ poor mental health
Roe *et al.* 2013, The UK [37]	Cross-sectional	33–55 y of socio-economically deprived areas	~100	PSS WEMWBS (short version)	Stress Well-being	Land-cover map	% GS at CAU	Increasing greenness ↓ stress^c^
Sarkar *et al.* 2013, The UK [38]	Cross-sectional	65–84 y	687	GHQ-30	Psychological distress	NDVI	500 m buffer	-
Adults (or population irrespective of age)								
Triguero-Mas *et al.* 2015, Spain [14]	Cross-sectional	34–64 y (physical activity, gender, degree of urbanization, socioeconomic status and social support)	8793	GHQ-12 SF-36	Perceived mental health	NDVI	100 m, 300 m, 500 m and 1 km buffers	Increasing greenness ↓ risk of poor mental health (for both tests and all buffers assessed)
Van den Berg *et al.* 2010, The Netherlands [39]	Cross-sectional	>18 y (stressful life events)	4529	GHQ-12	Perceived mental health	Land-cover map	% GS in 1 km & 3 km buffers	-
Weich *et al.* 2002, The UK [40]	Cross-sectional	Adults >16 y	1896	CES-D20	Depression	BESSC (audit)	Number of trees and amount of houses with private garden in the housing area	-
White *et al.* 2013, The UK [20]	Longitudinal	Adults	12818	GHQ-12	Mental health and well-being	Land-cover map	% GS at CAU	Increasing greenness ↓ risk of poor mental health

ADHD/DMS-IV: ADHD symptom Criteria of Diagnostic and Statistical Manual of Mental Disorder, 4th Edition; CAU level: Census area unit level; CES-D20: Centre for Epidemiologic Studies Depression scale (20 items); CIS-R: Revised Clinical Interview Schedule; DASS: Depression Anxiety and Stress Scales; GHQ-(12/30): General Health Questionnaire-(number of items included); GS: green space; ICD-10: International Classification of Disease; K(6/10): Kessler Psychological Distress Scale (number of items included); NDVI: Normalized Difference Vegetation Index; PSS: Perceived Stress Scale; SDQ: Strengths and Difficulties Questionnaire; SF-36: Short form health survey (36 items), WEMWBS: Warwick-Edinburgh Mental Well-being Scale; ^a^ SDQ measures hyperactivity, emotional symptoms, conduct problems, peer problems, prosocial behaviour and ADHD/DMS-IV measures inattention and hyperactivity-impulsivity symptoms; ^b^ A factor was created to define surrounding greenness. The factor included the presence of public green areas and the state of these areas, as well as other factors that did not have as much as weight as the first two within the factor; ^c^ These results were supported by objective measures of cortisol (biomarker of stress).

**Table 2:** Main characteristics and results of the studies on access to green spaces and mental health.

Author (Year, Country)	Study Design	Age of the Study Population (Stratifications/Interactions)	N	Tools to Measure Mental Health	Mental Health Item	Access to GS Indicator ^a^	Risk of Mental Health Problems
Exclusively children							
Amoly *et al.* 2014, Spain [30]	Cross-sectional	7–10 y	2111	SDQ ADHD/DSM-IV	Emotional & behavioural problems ^b^	Presence of a GS ≥0.05 km^2^ in a 300 m buffer	-
Balseviciene *et al.* 2014, Lithuania [28]	Cross-sectional	4–6 y (maternal education)	1468	SDQ	Emotional & behavioural problems ^b^	Distance to the nearest park of >1 ha and 65% of the land tree covered	Lower maternal education group: increasing distance ↑ behavioral problems, but not emotional problems
Markevych *et al.* 2014, Germany [29]	Cross-sectional	10 y (gender, urbanity degree)	1932	SDQ	Emotional & behavioural problems ^b^	Distance to the nearest GS	Increasing distance ↑ risk hyperactivity/inattention & peer relationship problems (after stratification only in males)
Adults (or population irrespective of age)							
Duncan *et al.* 2013, The USA [26]	Cross-sectional	~16 y (gender, ethnicity)	1170	MDS	Depression symptoms	Recreational open space & parks (400 m & 800 m buffers)	Increasing access to recreational open space in a 400 m buffer: ↓ risk in Asian Increasing access to parks in a 800 m buffer: ↑ risk in Black
Fan *et al.* 2011, The USA [27]	Cross-sectional	Adults 18–75 y	1544	PSS	Stress	Distance to the nearest park	-
Nutsford *et al.* 2013, New Zealand [23]	Ecological	>15 y	319521	Health ministry database	Anxiety/mood disorder treatment counts	Distance to total and useable GS	Increasing distance to usable GS ↑ risk of treatment
Adults (or population irrespective of age)							
Reklaitiene *et al.* 2014, Lithuania [41]	Cross-sectional	45–72 y (age, gender, park use)	7161	CES-D10	Depressive symptoms	Distance to the nearest park (of >1 ha and 65% of the land covered with green space;	Park users (≥4 h/week): increasing distance ↑ risk of depressive symptoms (after stratification only in females)
Sturm *et al.* 2014, The USA [42]	Cross-sectional	Adults	1070	MHI-5	Mental health	Distance to the studied parks (1.6 km)	Increasing distance ↓ mental health (no association beyond 1.6km)
Triguero-Mas *et al.* 2015, Spain [14]	Cross-sectional	34–64 y (physical activity, gender, degree of urbanization, socioeconomic status and social support)	8793	GHQ-12 SF-36	Perceived mental health	Presence of a GS within 100 m, 300 m, 500 m and 1 km buffers	-

ADHD/DMS-IV: ADHD symptom Criteria of Diagnostic and Statistical Manual of Mental Disorder, 4^th^ Edition; CES-D10: Centre for Epidemiologic Studies-Depression Scale (10 items); GS: green space; K6: Kessler Psychological Distress Scale (6 items); MDS: Modified Depression Scale; MHI-5: Mental Health Inventory (5 items); SDQ: Strengths and Difficulties Questionnaire; **^a^** All used land-cover map to calculate access to GS except Sturm *et al.* 2014, who used an audit tool; ^b^ SDQ measures hyperactivity, emotional symptoms, conduct problems, peer problems, prosocial behaviour and ADHD/DMS-IV measures inattention and hyperactivity-impulsivity symptoms.

### 3.1. Surrounding Greenness

Of the 28 articles included in the systematic review, 22 evaluated the mental health benefits of surrounding greenness. In most of the studies surrounding greenness was measured as the percentage of green space in a specific buffer (from 300 m to 3 km) or at census area unit level (CAU) using a land-cover map (Table 1). Moreover, seven studies used the Normalized Difference Vegetation Index (NDVI) as indicator of surrounding greenness located in buffers of 100 to 800 m or at CAU [14,27,28,30,33,38].

Four studies from different countries limited their study population to children of ages between 3 and 10 years [19,28,29,30] and out of these only one study observed that increasing surrounding greenness was associated with less emotional and behavioural problems [30]. A longitudinal study observed that this association only occurred in children from low-income families [19], whereas another cross-sectional study reported increased behavioural problems with increasing surrounding greenness in children from mothers with a higher education [28]. Markevych *et al.* did not find any association [29] (Table 1). Based on these studies, we classified the evidence of causal relationship between surrounding greenness and emotional and behavioural problems in children as inadequate.

Regarding the 18 studies including adults (or population irrespective of age), most of these except five [24,27,38,39,40] observed a reduced risk of poor mental health or other related disorders with increasing surrounding greenness (Table 1), including the three longitudinal studies available; Alcock *et al.* showed that after three years of moving to a greener area the mental health of the participants had improved compared to those that moved to less green area [22]. White *et al.* also observed a small reduction of mental health problems with increasing greenness [20]. An Australian study, including more than 65,000 participants, observed that the mental health benefits of surrounding greenness was not linearly associated with increasing greenness and that the results differed by age and gender [18] (Table 1). Overall, we classified the evidence of causal relationship between surrounding greenness and mental health and related disorders in adults as limited.

### 3.2. Access to Green Spaces

Eight cross-sectional studies [14,26,27,28,29,30,41,42] and one ecological study [23] evaluated the mental health benefits of access to green spaces. Access to green spaces was mainly measured as the distance to the nearest green space, park or public open space [23,27,28,29,41,42]. Two studies included parks with a minimum size [28,41] and one differentiated between total and usable green space [23]. Three studies used a dichotomous classification to evaluate access to green spaces. The first study classified study participants according to the presence of green spaces of >0.05 km^2^ in a 300 m buffer [30], the second study followed a similar approach but did not restrict the size of green spaces [14], and the last study classified study participants based on the density of recreational open space and parks within buffers of 400 and 800 m [26] (Table 2).

Three studies from different countries limited their study population to children of ages between 4 and 10 years [28,29,30]. In two of the studies increasing distance from the green space increased the risk of behavioural, but not emotional problems. However, in one of them these associations only occurred in children from mothers with low education [28] and in the other one the associations were stronger in males [29]. The study of Amoly *et al.* assessed access to green spaces of >0.05 km^2^ using the recommended distance of the European commission (300 m) and did not find any association [30] (Table 2). Because of the limited number of studies we classified the evidence of a causal relationship between access to green spaces and emotional and behavioural problems in children as inadequate.

In adults, three studies observed that increasing distance to the nearest green space or park increased the risk of mental health problems [42], depressive symptoms [41] and treatment for anxiety and mood disorders in adults [23]. However, Fan *et al.* did not find an association between the distance to the nearest park and stress [27]. Finally, Duncan *et al.* observed a couple of statistically significant associations between density of recreational open spaces and parks in buffers of 400 and 800 m and depression symptoms in teenagers; whereas the risk of depression was increased for Blacks, the risk was decreased for Asians. No associations were observed for the general population or other ethnicities [26] (Table 2). Triguero-Mas *et al.* did not find associations either between access to green spaces, defined as the presence of green spaces in a 300 m buffer, and mental health [14]. Overall, we classified the evidence of a causal relationship between access to green spaces and mental health and related disorders in adults as inadequate.

### 3.3. Quality of Green Spaces

We identified two studies assessing the mental health benefits of the quality of green spaces, which was evaluated using different non-validated audit tools based on subjective judgment [17,43]. In the longitudinal study green spaces were defined as serene, wild, lush, spacious or culture. Access to serene and spacious green spaces was associated with a reduced risk of poor mental health (measured with the GHQ) in women who were physically active [17]. The cross-sectional study took into account different characteristics of the green spaces in the street to define better quality, which was associated with a better mental health (measured with the MHI). This study included quantity of green space to adjust the models, but this variable was poorly defined [43].

### 3.4. Blue Spaces

Only three cross-sectional studies evaluated the mental health benefits of blue spaces [14,21,34]. The first study observed that the percentage of blue spaces (fresh and salt water surface) in buffers of 1 and 3 km was not associated with mental health [34]. The second study observed that living less than 5 km from the coast improved mental health (measured with the GHQ) compared to living further away, even after adjusting for percentage of green space and fresh water [21]. And the third study did not observe associations between the presence of blue spaces (within buffers ranging from 100 to 1000 m) and mental health [14]. Finally, one study could not assess the effects of blue spaces on children’s behaviour and emotion because less than 2% of the study population lived within 500 m of the beach [30]. We classified evidence of causal relationship as inadequate.

## 4. Discussion

In this review based on objective and/or standardized measures of both green and blue spaces and mental health outcomes, we found limited evidence of mental health benefits of long-term residential surrounding greenness in adults. For access to green space and for studies in children the evidence was inadequate. The main limitations of the present review were the limited number of studies available and the heterogeneity across studies regarding green space assessment.

### 4.1. Green and Blue Spaces Definitions and Indicators and Mechanisms

Currently there is not a standardized approach to define exposure to green (or blue) space, or to define what we actually mean by surrounding greenness or access to green space, concepts that sometimes can also overlap. This is actually reflected in the diverse definitions provided by each of the studies included in the present review. Moreover, there are no recommendations of which green or blue spaces indicators are better to use and there is not a consistent use of them. For example, the advantage of using indices such as the NDVI is that the level of greenness measured by the different studies is always comparable. However, other measurements such as the percentage of greenness based on land-cover maps might vary across studies when different criteria are chosen to define green space (*i.e.*, inclusion or exclusion of private gardens, exclusion of green spaces smaller than a certain size, inclusion of the total or the usable green space, *etc.*) and therefore results and conclusions might also differ. For example, the associations observed by Markevych *et al.* between increasing distance to green space and increasing behavioural problems in children disappeared once green spaces smaller than 5000 m^2^ were excluded from the analysis [29]. This is a clear example on how different definitions might lead to different conclusions.

Also, there are recommendations on the distance between residence and the nearest open public space despite it is not widely accepted yet. The current recommended distance between residence and the nearest open public space is 300 m [8]. This recommendation might be supported by the fact that 300–400 m is the threshold after which use of green spaces starts to quickly decline [17]. However, some studies suggest that people are willing to walk even longer distances to access green areas [44,45]. Furthermore, in three studies of the present review beneficial effects of surrounding greenness were observed in buffers of even 3 km or areas above 5 km^2^ (CAU) [23,25,34].

Various mechanisms have been suggested to explain the mental health benefits attributed to green and, in a lesser extent, blues spaces. These mechanisms include: (a) intrinsic qualities of green and blue spaces that enhance health or well-being (restoration theory) and that have an effect through simple viewing or observing green or blue spaces; (b) the healthy environment associated with green spaces (less temperature, air pollutants and noise have been observed in greener areas [46,47,48,49]) and (c) the opportunity to perform physical activity and (d) to enhance social interactions [5,50]. In this sense, and depending on the actual mechanism or the set of mechanisms that would explain the association between greenness and mental health, different results with different types of green space could be expected. Additionally, studies should take into account aspects that would provide more accurate results and therefore more refined information than what has been done so far. For example, if the beneficial associations of green spaces on mental health are through a restoration effect of viewing or enjoying green spaces, then, people using green spaces are probably more benefitted from living near (and therefore having an easier access) to green spaces than those who do not make use of them [30,41]. Another aspect is the type of use of these green spaces; a study included in the present review observed that the reduced psychological distress associated with living near green spaces occurred mainly in those adults who were physically active [18]. These results indicate that it is important to include additional information in future studies, not only on the use of green spaces and the type of activities performed, but also on the motivations to use these areas [51]. For instance, it could be that the use of green spaces or the mental health benefits of green spaces are influenced by their quality and characteristics [52] or by how these spaces are perceived. Perceptions can vary according to the culture, the age, or other determinants of the studied subjects and therefore this needs to be taken into account [53]. In this sense, there are number of studies that evaluate exposure to green spaces using the perception of the study participants [51,54,55,56]. However, these were not included in the present review because we wanted to focus on studies with objective measures and provide information based on epidemiological evidence that could be used for health impact assessment.

Furthermore, other aspects of the built environment (e.g., degree of urbanization or ease of accessibility) could influence the use of green spaces and explain indicators such as the NDVI. Unfortunately, this aspect has been poorly assessed in the current literature [50,57,58]. In fact, in the present review quality of green spaces has only been evaluated in a couple of studies which used non-standardized audit tools [17,43]. Currently, international efforts are being undertaken in order to provide tools of comparability for different items of the built environment between countries [58]. Furthermore, new technological tools such as Google Street or Google Earth, together with audits and the use of smartphones, which currently can provide lots of different information, could help epidemiological studies to create an objective and standardize tool to perform validation studies and define quality of both green/blue spaces and other aspects of the built environment [59,60,61]. To widen the knowledge on build environment influences on green spaces would facilitate the understanding of the link between quantity and quality.

### 4.2. Mental Health Assessment

In the present review all studies including children assessed behavioural and emotional problems with the same tool (SDQ), which is a valid general screening tool to evaluate behaviour in children. The fact that all studies used it facilitates comparability between studies. However, other tests available would also be valid and probably more complete and refined, such as the Child Behavior Checklist (CBCL), a test for children between 2 and 18 years assessing internalizing (*i.e.*, anxious, depressive, and overcontrolled) and externalizing (*i.e.*, aggressive, hyperactive, noncompliant, and undercontrolled) behaviours [62], or the Behavior Assessment System for Children-2 (BASC-2), a multiple informant based questionnaire designed to assess a broad range of emotional and behavioral symptomatology seen in youth. The BASC-2 assesses common child mental health concerns including depression, anxiety, conduct problems, and attention difficulties [63]. Future studies should also include neurocognitive tests, as so far none of the published studies includes such information. In adults, different approaches were used to define the “mental health status” of the participants, which might differ also according to the aim of the study: evaluation of the general mental health or evaluation of certain related disorders such as depression, anxiety, stress or distress. For the evaluation of the general mental health, the GHQ is a validated and easy to use tool and actually one of the most used tests. Therefore, future studies aiming to evaluate general mental health should include GHQ to facilitate comparability and further meta-analysis between studies. However, other tests might also be considered, such as the Warwick-Edinburgh Mental Well-being Scale (WEMWBS) [64], as it may be more appropriate if interested in the role of green and blue space in enhancing positive wellbeing.

### 4.3. Mental Health Determinants

As mentioned, physical activity is an important determinant of mental health, however, other factors such as age, gender, education and socioeconomic position are also strong determinants [1]. Some studies included in the present review observed that the health benefits of green spaces could be modified by these variables [18,19,25,28,34,37]. For instance, in a study conducted in The UK the risk of emotional problems was reduced in relation to surrounding greenness in poor children of 3 to 5 years of age, but not in children from a better social class [19]. Another longitudinal study observed non-linear associations between mental health and surrounding greenness according to the age and gender of the participants [18]. Overall, it seems that individuals from lower socioeconomic positions are more susceptible to benefit from living near green areas; if further evidence shows such benefits in individuals at higher risk of suffering from mental health problems, then promotion of green spaces in more deprived areas could be a way to reduce existing health inequalities in cities [65].

### 4.4. Limitations of Our Classification Criteria

Scoring the quality of the studies and classifying the evidence can have a degree of subjectivity. In the present review, in order to reduce such subjectivity, two independent reviewers, with the help of a third reviewer, conducted the scoring of the quality of the studies and classified the evidence.

We should also consider that classification of the evidence could be affected by publication bias. In this sense, those studies with significant associations would be more prone to report the results obtained. Nevertheless, the evidence of an association between green spaces and better mental health is still limited and results obtained by the different existing studies often depended on aspects such as the gender, the social class, the physical activity, *etc.* Additionally, quite a few studies were at risk of obtaining significant results due to multiple testing, although some of them did not even obtain significant associations after multiple analyses [17,20,21,23,24,26,27,30,34,37,40]. Two methodological aspects to take into account of the studies included in this review is that some of them did not exclude participants that had lived less than a year in their residence at the time the study was conducted. This somehow limits the inference of long-term effects of green spaces if the exposure has occurred for a short period of time until outcome assessment. Also, it is important to take into account that some studies used one single measurement of greenness that was applied equally across several years of study, with no corrections for changes within areas over time. These are issues that future studies should address when possible.

Finally, due to our restrictive inclusion criteria, in the present systematic review we excluded many experimental, qualitative and observational studies that also evaluated the beneficial health effects of green and blue spaces. This somehow limits our capability of capturing a broader picture of the evidence so far. However, it also provides more consistent epidemiological evidence-based of the effects of long-term exposure to green and blue spaces, which were the associations we were interested in.

### 4.5. Limitations for Conducting a Meta-Analysis

Based on the main results of the studies included in the present work (Table 3), we tried to perform a meta-analysis of the association between surrounding greenness and mental health in adults, as this was the exposure-outcome combination for which we had more studies available. However, most studies did not provide all the information needed to conduct the meta-analysis and we could not obtain extra information from all the corresponding authors that were contacted via e-mail. Some studies provided limited descriptive information and others only provided the estimate of the regression analyses but not the confidence interval-preferable-or the standard deviation (SD). This made difficult the transformation of the estimates to allow the performance of the meta-analysis [66]. In order to facilitate future meta-analyses and provide clear information to policy makers, further studies should provide the estimates and the confidence intervals of the main analyses, as well as descriptive information including the mean or median and the interquartile range (IQR) or SD for continuous exposures/outcomes as well as the percentage for categorical exposures/outcomes.

## 4. Conclusions

According to the World Health Organization, mental health promotion should include actions that create living conditions and environments that support mental health and allow people to adopt and maintain healthy lifestyles [67]. Given the increase in mental health problems and the ongoing massive urbanization, especially in developing countries, results from the present review, which showed limited evidence of long-term beneficial mental health effects of surrounding greenness, should be taken into account in future urban planning. However, we need further research and more detailed information on the characteristics of the green and blue spaces that promote better mental health (quantity, quality and distance) and the mechanisms, which are highly related to the use of these spaces. Future studies should also include stratified analyses according to social class, education, age and gender, as they possibly could modify the beneficial health effects of green and blue spaces. In terms of comparability, future studies are recommended to use NDVI as marker of surrounding greenness or use a well- established definition of percentage of green space. Regarding the evaluation of access to major green spaces (≥0.05 km^2^) studies should conduct sensitivity analyses using other distances than the current recommended distance (300 m), as there is no evidence that this distance is actually determinant for the beneficial health effects of green spaces. There is still a big debate on whether studies should use the Euclidian or the network distances to evaluate access to green spaces; the first is used in most guidelines and studies and is easier to calculate. However, the second might be more realistic in relation to walked distances and the ease of access. As there is not a consensus yet, we propose to evaluate both where possible.

Finally, it would be interesting in future studies to adjust the models assessing residential greenness and mental health for the greenness at school (children) and at work (adults), as we daily spend an important part of our time in these places. Regarding mental health assessment, the GHQ seems to be a good tool for adults, whereas for children SDQ, in terms of comparability, but also CBCL or BASC-2 are appropriate tools.

**Table 3:** Main estimations of the association between surrounding greenness or access to green space and mental health ^a^ (results presented by exposure type, children/adults and then outcome type) ^a^.

Author (Year)	N	Exposure Type	Exposure Description	Questionnaire/Outcome	Estimate Type	Estimate ^b^
**Surrounding greenness**						
***Children***						
Amoly *et al.* 2011 [30]	2111	NDVI in 500 m buffer	Mean (IQR) = 0.06 (0.05)	SDQ (cont.)	% change (95%CI)	−4.0 (−6.7, −1.2)
Balseviciene *et al.* 2014 [28]	1172 (high maternal education)	NDVI in 300 m buffer	Not provided	SDQ (cont.)	β	2.29 (*p* < 0.1)
	296 (low maternal education)					1.29 (*p* > 0.1)
Flouri *et al.* 2014 [19]	6384	% GS at CAU	Range = 0 to 97%	SDQ (cont.) ^c^	β (SE)	0.00 (0.01)
Markevych *et al.* 2014 [29]	1932	NDVI in 500 m buffer	Not provided	SDQ (cont.)	Data not shown (no association)	
***Adults (or population irrespective of age)***						
*General mental health*						
Alcock *et al.* 2014 [22]	1064	See footnote ^d^	-	GHQ-12 (cont.)	β (SE)	0.431 (0.162) ^b^
Araya *et al.* 2007 [31]	3870	Factor of “green spaces” ^e^	Mean (SD) = 0.97 (0.77)	CIS-R (cont.)	β (95%CI)	−0.01 (−0.09, 0.06)
Astell-Burt *et al.* 2014 ^f^ [18]	29,626 (men)	% GS at CAU	The highest tertile	GHQ-12 (cont.)	β (SE)	−0.33 (0.12)
	35,781 (women)					0.09 (0.13)
De Vries *et al.* 2003 [34]	10,197	% GS between 1 and 3 km	Not provided	GHQ (dich.)	β (SD)	−0.01 (0.003)
Maas *et al.* 2009 [35]	10,089	% GS in 3 km	Mean (SD) = 60.7 (21.6)	GHQ-12 (dich.)	β (SE)	−0.004 (0.002)
Richardson *et al.* 2013 [36]	8157	% GS at CAU	The highest quartiles	SF-36 (dich.)	OR (95%CI)	0.81 (0.66, 1.00)
Sarkar *et al.* 2013 [38]	687	NDVI	Mean (range) = 0.09 (−0.06, 0.33)	GHQ-30 (dich.)	OR (95%CI)	0.79 (0.52, 1.23)
Triguero-Mas *et al.* 2015 [14]	8793	NDVI in 300 m	Median (1st, 3rd quartile) = 0.16 (0.13, 0.21)	GHQ-12 (dich.)	OR (95%CI)	0.79 (0.71, 0.88)
				SF-36 (dich.)	OR (95%CI)	0.90 (0.83, 0.98)
Van den Berg *et al.* 2010 [39]	4529	% GS in 3 km	dich. (<62.82% GS)	GHQ (cont.)	β (z)	0.00 (0.03)
White *et al.* 2013 [20]	12,818	% GS at CAU	Mean (SD) = 64.6 (16.7)	GHQ-12 (cont.)	β (SE)	−0.004 (0.01)
*Stress/distress*						
Astell-Burt *et al.* 2013 [32]	260,061	% GS in 1 km buffer	The highest quintile	K10 (dich.)	OR (95%CI)	0.91 (0.84, 1.00)
Fan *et al.* 2011 ^g^ [27]	1544	NDVI (0-10 scale)	Mean (SD) = 3.6 (0.75)	PSS (cont.)	β (95%CI)	−0.04 (−0.10, 0.01)
Francis *et al.* 2012 ^h^ [24]	911	Number of POS (≥5 to 14 )	44%	K6 (cont.)	OR (95%CI)	1.43 (0.96, 2.13) ^b^
Roe *et al.* 2013 [37]	103	% GS at CAU	Not provided	PSS (cont.)	β (95%CI)	−0.08 (−0.14, −0.01)
				WEMWBS (cont.)		Not provided
*Clinical outcomes (depression, anxiety, mood disorders* )						
Araya *et al.* 2007 [31]	3870	Factor of “green spaces” ^e^	Mean (SD) = 0.97 (0.77)	ICD-10 (dich.)	OR (95%CI)	0.94 (0.90, 0.99)
Beyer *et al.* 2014 [33]	2479	% Tree canopy	Mean (SD) = 0.17 (0.18)	DASS (cont.)	β (SE)	−4.02 (1.17)
Maas *et al.* 2009 [25]	345,143	% GS in 3 km	Mea*N* = 60.8%	Primary care records (depression-dich.)	OR (95%CI)	0.98 (0.96, 1.00)
Nutsford *et al.* 2013 [23]	319,521	% GS in 3 km	Mea*N* = 16%	Anxiety/depression treatment (dich.)	IRR (95%CI)	0.96 (0.94, 0.97)
Weich *et al.* 2002 [40]	1896	>5 trees around the house	18.2%	CES-D20 (dich.)	OR (95%CI)	1.20 (0.78, 1.84)
**Access to green spaces**						
***Children***						
Amoly *et al.* 2011 [30]	2111	GS > 0.05 km^2^ within 300 m	18.1%	SDQ (cont.)	% change (95%CI)	−1.3 (−8.2, 6.2)
Balseviciene *et al.* 2014 [28]	1172 (high maternal education)	Distance to the nearest park	Mean (SD) = 667 (544)	SDQ (cont.)	β	−0.01 (*p* > 0.1)
	296 (low maternal education)					0.07 (*p <* 0.05)
Markevych *et al.* 2014 [29]	1932	Distance to the nearest GS	Median (IQR) = 289.1 (368.1)	SDQ (dich.)	OR (95%CI)	1.07 (0.92, 1.23)
***Adults (or population irrespective of age)***						
Duncan *et al.* 2013 [26]	1170	Park density in 400 m	Not provided	MDS (cont.)	β (SE)	−0.002 (0.05)
Fan *et al.* 2011 [27]	1544	Distance to the nearest park (m)	Mean (SD) = 0.24 (0.18)	PSS (cont.)	β (95%CI)	0.024 (−0.24, 0.28)
Nutsford *et al.* 2013 [23]	319,521	Distance to the GS (km)	Mea*N* = 0.198	Anxiety/depression treatment (dich.)	IRR (95%CI)	1.26 (0.95, 1.68)
Reklaitiene *et al.* 2014 [41]	7161	Living > 1 km from the nearest park	≈25%	CES-D10 (dich.)	OR (95%CI)	0.96 (0.71, 1.29)
Sturm *et al.* 2014 [42]	1070	Distance to parks of interest	Cut-offs, no further info	MHI-5 (cont.)	β (SE)	−0.33 (1.17) ^b^
Triguero-Mas *et al.* 2015 [14]	8793	Presence of a GS within 300 m	60.3%	GHQ-12 (dich.)	OR (95%CI)	0.93 (0.79, 1.09)
				SF-36 (dich.)	OR (95%CI)	0.95 (0.83, 1.08)

CES-D(10/20): Centre for Epidemiologic Studies-Depression Scale (number of items included); cont.: outcomes treated as a continuous variable; K6: Kessler Psychological Distress Scale (6 items); MDS: Modified Depression Scale; MHI-5: Mental Health Inventory (5 items); CAU level: Census area unit level; DASS: Depression Anxiety and Stress Scales; dich.: outcome treated as a dichotomized variable; GHQ-(12/30): General Health Questionnaire-(number of items included); GS: green space; ICD-10: International Classification of Disease; K(6/10): Kessler Psychological Distress Scale (number of items included); NDVI: Normalized Difference Vegetation Index; PSS: Perceived Stress Scale; SDQ: Strengths and Difficulties Questionnaire; SF-36: Short form health survey (36 items), WEMWBS: Warwick-Edinburgh Mental Well-being Scale; ^a^ Some of the data was not available in the manuscripts and was obtained from the corresponding authors; ^b^ For all continuous outcomes a higher score indicates worse mental health or more behavioural problems, except the study of Alcock *et al.* 2014 in which the GHQ score was inversed and the study of Sturm *et al.* 2014 in which a higher score indicates better mental health. For all dichotomous outcomes (0/1) 1= worse mental health, more behavioral problems or being more prone to having a psychiatric problem. The study of Francis *et al.* 2012 calculated the odds of low psychological distress instead of the odds for high psychological distress; ^c^ The association with the total SDQ score was not evaluated and thus the association with conduct problems is reported (results were similar for the other SDQ items); ^d^ This study estimated mental health changes in two populations; those moving to greener areas and those moving to less green areas; ^e^ A factor was created to define surrounding greenness. The factor included the presence of public green areas and the state of these areas, as well as other factors that did not have as much as weight as the first two within the factor; ^f^ Non-linear associations according to age and gender; ^g^ Outcome log-transformed; ^h^ Results of the non-adjusted model as the adjusted model results were unavailable.

Nevertheless, the test chosen should suit the aims of the study and be the appropriate to evaluate the outcomes of interest. Longitudinal analyses are also needed to prove causal inference, as well as studies that look at the influence of shifting circumstances and characteristics through the life course and on the capacity/inclination of individuals to use and benefit from their local green/blue spaces. In the current review only one study included cortisol as a biomarker of stress [37], but it would be interesting to include it in future studies in order to be able to further explain and understand the associations found between green (and blue) spaces and mental health. Finally, we need further evidence of the mental health benefits of green and blue spaces from studies conducted in different countries with different characteristics in terms of climate, living conditions and culture. For instance, in countries with bad sanitary conditions, the fact of living near green or blue areas can be negative for health, including mental health, as in these areas there is a higher risk of communicable diseases [68]. In this sense, low- and middle-income countries, where the urban growth is evolving faster and with a greater impact than in high-income countries and where mental health is a largely undervalued problem which is only now beginning to emerge [1], should be the next settings in which to explore the potential health benefits of green and blue spaces.

## Supplementary Files

Supplementary File 1
