# Supplementary material for: Mental Health Benefits of Long-Term Exposure to Residential Green and Blue Spaces: A Systematic Review
Source: Int J Environ Res Public Health. 2015 Apr 22;12(4):4354–79. doi: 10.3390/ijerph120404354 (PMC4410252; doi:10.3390/ijerph120404354)
Supplement: Supplementary File 1 [file ijerph-12-04354-s001.pdf]

Supplementary Information

Mental Health Benefits of Long-Term Exposure to Residential Green and Blue Spaces: A Systematic Review

---

Table S1. Additional characteristics and quality scores of the studies included in the systematic review on green and blue spaces and mental health — Page S2

Table S2. Final criteria for quality assessment of the studies — Page S7

Table S3. Specific scores for each item evaluated and the final quality scores and categories given to each study — Page S8

Table S1. Additional characteristics of the studies included in the systematic review on green and blue spaces and mental health.

Author (Year, Country)	Study Design/Population (N)	Statistical Methods	Co-Variables of Adjustment and Interactions Evaluated	Other Information
Alcock *et al.* 2014, The UK [1]	Longitudinal Adults (*N* = 1064)	Fixed effects regression	✓ CAU level: income, employment and education deprivation and crime rate index<br>✓ Individual level: age, education, marital status, living with children, household income, work-limiting illness, labour market status, residence type and commuting time	✓ It does not evaluate quality or use of GS<br>✓ Participants have been living at least 1 year in the studied residence<br>✓ Restricted to urban areas from England<br>✓ Risk of lost-to-follow up of those with worse mental health<br>✓ Gardens included
Amoly 2014 et al., Spain [2]	Cross-sectional Children 7–10 y (*N* = 2111)	Quasi-Poisson mixed effects model	✓ CAU level: socioeconomic status<br>✓ Individual level: gender, school level, ethnicity, preterm birth, breastfeeding, exposure to environmental tobacco smoke, maternal smoking during pregnancy, responding person, parental education, employment and marital status	✓ It does not evaluate quality of GS<br>✓ It takes into account use of GS<br>✓ No mention of the minimal time of residence<br>✓ School greenness evaluated<br>✓ Restricted to urban areas
Annerstedt *et al.* 2012, Sweden [3]	Longitudinal Adults 18–80 y (*N* = 9230)	Logistic regression	✓ Mental health of the first follow-up, age, financial stress, cohabitation status, country of origin<br>✓ Interaction with physical activity	✓ It evaluates quality of GS<br>✓ Exclusion of people who changed residence<br>✓ Individuals living in larger city centres excluded<br>✓ Responders slightly higher education<br>✓ Lost-to-follow up of those with worse mental health
Araya *et al.* 2007, Chile [4]	Cross-sectional Adults 16–64 y (*N* = 3870)	Multilevel linear/logistic regression	✓ CAU level: episodes of violent crime reported to local police and general quality, facilities, and empty sites of the CAU<br>✓ Individual level: age, gender, presence of disease, income, education, marital status, housing type, number of supportive individuals, alcohol use	✓ Exposure includes presence of public green areas and its state by creating a factor that includes both<br>✓ It does not evaluate use of GS<br>✓ No mention of the minimal time of residence<br>✓ Socially deprived individuals less likely to participate<br>✓ GS evaluated 4y after mental health assessment

Table S1. *Cont.*

Author (Year, Country)	Study Design/Population (N)	Statistical Methods	Co-Variables of Adjustment and Interactions Evaluated	Other Information
Astell-Burt *et al.* 2013, Australia [5]	Cross-sectional >45 y (*N* = 260,061)	Multilevel logit regression	✓ CAU level: socioeconomic index of the studied areas, urban vs remote areas<br>✓ Individual level: social interactions, age, gender, ancestry, country of birth, language spoken at home, household income, education, economic status, couple status, smoking, alcohol consumption, BMI<br>✓ Interaction with physical activity	✓ It does not evaluate quality of GS<br>✓ No mention of the minimal time of residence<br>✓ Agriculture land and private gardens excluded
Astell-Burt *et al.* 2014, The UK [6]	Longitudinal >15 y (*N* = 65,407)	Multilevel linear regression	✓ Age, gender, employment status, household tenure, marital status, education, marital status, smoking, household income<br>✓ Interaction with age and gender	✓ It does not evaluate quality or use of GS<br>✓ Participants have been living at least 1 year in the studied residence<br>✓ GS change over time taken into account<br>✓ Only people living in urban neighbourhoods<br>✓ Private gardens excluded
Balseviciene *et al.* 2014, Lithuania [7]	Cross-sectional Children 4–6 y (*N* = 1468)	Non-hierarchical linear regression	✓ Age, gender, parenting stress<br>✓ Interaction with maternal education	✓ It does not evaluate quality or use of GS<br>✓ No mention of the minimal time of residence<br>✓ Only children from urban areas
Beyer et al. 2014, The USA [8]	Cross-sectional 21–74 y (*N* = 2479)	Linear regression	✓ CAU level: urbanicity, unemployment, instability, poverty, population density, education, housing tenure, % Afro-American, household income.<br>✓ Individual level: age, gender, ethnicity, education, income, marital status and insurance status, length of residence in the neighbourhood	✓ It does not evaluate quality or use of GS<br>✓ No mention of the minimal time of residence
De Vries *et al.* 2003, The Netherlands [9]	Cross-sectional All ages (*N* = 10,197)	Logistic multilevel analysis	✓ CAU level: urbanity<br>✓ Individual level: age, gender, education, number of rooms, type of health insurance, number of life-events<br>✓ Interaction with education and urbanity degree	✓ It does not evaluate quality or use of GS<br>✓ Participants have been living at least 1 year in the studied residence<br>✓ Exclusion of those with changes in urbanity in their neighbourhood<br>✓ GS and health data collected at different moments

Table S1. *Cont.*

Author (Year, Country)	Study Design/Population (N)	Statistical Methods	Co-Variables of Adjustment and Interactions Evaluated	Other Information
De Vries *et al.* 2013, The Netherlands [10]	Cross-sectional<br>All ages<br>(*N* = 1641)	Multilevel analysis	✓ Individual level: gender, age, education, income, life events, children at home, smoking, excessive drinker<br>✓ Mediation of stress, social cohesion and green and physical activity	✓ Evaluates quality of GS<br>✓ Partially evaluates use of GS<br>✓ No mention of the minimal time of residence<br>✓ Neighbourhoods with peculiar or extreme socioeconomic profiles excluded
Duncan *et al.* 2013, The USA [11]	Cross-sectional<br>Adolescents ~16 y<br>(*N* = 1170)	Ordinary least squares regression	✓ CAU level: school, % Black & Hispanics, households below poverty, % born outside the buffer<br>✓ Individual level: race/ethnicity, gender, age, nativity, family structure<br>✓ Interaction with gender and ethnicity	✓ It does not evaluate quality or use of GS<br>✓ No mention of the minimal time of residence
Fan *et al.* 2011, The USA [12]	Cross-sectional<br>Adults 18–75 y<br>(*N* = 1544)	Linear regression	✓ Individual level: gender, age, ethnicity, education, household income, employment status, marital status, number of children, physical activity, social support	✓ It does not evaluate quality or use of GS<br>✓ No mention of the minimal time of residence<br>✓ Years that participants have been living in the area
Flouri *et al.* 2014, The UK [13]	Longitudinal<br>Children 3 & 7 y<br>(*N* = 6384)	Mixed model	✓ CAU level: deprivation<br>✓ Individual level: age, gender, ethnicity, socio-economic status, adverse life events, maternal education, marital status parents, garden access<br>✓ Interaction with socioeconomic status	✓ It does not evaluate quality of GS<br>✓ No mention of the minimal time of residence<br>✓ Exclusion of private gardens<br>✓ Rural areas excluded
Francis *et al.* 2012, Australia [14]	Cross-sectional<br>Adults 20–79 y<br>(*N* = 911)	Logistic regression	✓ CAU level: crime (self-reported), socioeconomic status<br>✓ Individual level: gender, age, marital status, children at home, education, work status, hours worked, BMI, life events, participation in social groups, social network and support, sense of community	✓ Evaluates quality and use of GS<br>✓ Participants have been living at least 1 year in the studied residence
Maas *et al.* 2009, The Netherlands [15]	Cross-sectional<br>12 to >65 y<br>(*N* = 4842–10,089)	Multilevel logistic regression	✓ CAU level: urbanicity<br>✓ Individual level: age, gender, household size, education, household income<br>✓ Mediation analyses with social support	✓ It does not evaluate quality or use of GS<br>✓ No mention of the minimal time of residence<br>✓ Gardens and small GS excluded
Maas *et al.* 2009, The Netherlands [16]	Cross-sectional<br>All ages (*N* = 345,143)	Multilevel logistic regression	✓ CAU level: urbanicity<br>✓ Individual level: age, gender, education, health insurance, work situation<br>✓ Interaction with age, socioeconomic status, urbanicity	✓ It does not evaluate quality or use of GS<br>✓ Participants have been living at least 1 year in the studied residence<br>✓ Small GS excluded if not predominant

Table S1. *Cont.*

Author (Year, Country)	Study Design/Population (N)	Statistical Methods	Co-Variables of Adjustment and Interactions Evaluated	Other Information
Markevych *et al.* 2014, Germany [17]	Cross-sectional 10 y (*N* = 1932)	Logistic regression	✓ Individual level: age, gender, parental education, maternal age at birth, civil status, time in front of a screen, time spent outdoors ✓ Interaction with gender and urbanicity ✓ Mediation analysis: physical activity	✓ It does not evaluate quality or use of GS ✓ Sensitivity analyses excluding GS >5000m^2 ✓ Participants have been living at least 1 year in the studied residence
Nutsford *et al.* 2013, New Zealand [18]	Ecological >15 y (*N* = 319,521)	Negative binomial regression	✓ CAU level: deprivation levels (derived from nine variables)	✓ It does not evaluate quality or use of GS ✓ No mention of the minimal time of residence
Reklaitiene *et al.* 2014, Lithuania [19]	Cross-sectional 45–72 y (*N* = 7161)	Logistic regression	✓ Individual level: age, marital status, education, smoking, use of alcohol, BMI ✓ Interaction with age, gender, park use	✓ It does not evaluate quality of GS ✓ Evaluates use of GS ✓ No mention of the minimal time of residence
Richardson *et al.* 2013, New Zealand [20]	Cross-sectional >15 y (*N* = 8157)	Multilevel logistic regression	✓ Individual level: gender, age, smoking, index of socio-economic deprivation ✓ Interaction with physical activity	✓ It does not evaluate quality or use of GS ✓ No mention of the minimal time of residence ✓ Rural areas excluded
Roe *et al.* 2013, The UK [21]	Cross-sectional 33–55 y (*N* = ~100)	Linear regression	✓ Individual level: age, gender, deprivation level, access to gardens	✓ It does not evaluate quality or use of GS ✓ Participants have been living at least 1 year in the studied residence ✓ Non-working adults from socio-economically deprived areas ✓ Private gardens excluded
Sarkar *et al.* 2013, The UK [22]	Cross-sectional 65–84 y (*N* = 687)	Multilevel logistic regression	✓ CAU level: deprivation ✓ Individual level: age, alcohol consumption, social class, education, chronic vascular comorbidities	✓ Partial evaluation of quality of GS ✓ It does not evaluate use of GS ✓ No mention of the minimal time of residence
Sturm *et al.* 2014, The USA [23]	Cross-sectional Adults (*N* = 1070)	Hierarchical linear regression	✓ Individual level: age, gender, BMI, overall health status, unemployment ✓ Mediation analysis: physical activity, park frequency	✓ It does not evaluate quality of GS ✓ Evaluates use of GS ✓ No mention of the minimal time of residence ✓ Seasonal effects and regional unemployment rates assessed

Table S1. *Cont.*

Author (Year, Country)	Study Design/Population (N)	Statistical Methods	Co-Variables of Adjustment and Interactions Evaluated	Other Information
Triguero-Mas *et al.* 2015, Spain [24]	Cross-sectional 34–64 y (*N* = 8793)	Logistic regressions	✓ Individual level: gender, age, education level, birth place, type of health insurance, marital status, and indicators of household and neighbourhood socioeconomic status. Degree of urbanization as an effect modifier.<br>✓ Mediation analysis: social support, physical activity	✓ It does not evaluate quality of GS<br>✓ It does not evaluate use of GS<br>✓ No mention of the minimal time of residence
Van den Berg *et al.* 2010, The Netherlands[25]	Cross-sectional >18 y (*N* = 4529)	Multilevel linear regression	✓ CAU level: level of urbanity<br>✓ Individual level: age, gender, education, income<br>✓ Interaction with physical activity stressful life events	✓ It does not evaluate quality or use of GS<br>✓ Participants have been living at least 1 year in the studied residence<br>✓ Interviews performed across the four seasons<br>✓ Small GS excluded
Weich *et al.* 2002, The UK [26]	Cross-sectional Adults >16 y (*N* = 1896)	Linear and logistic regression	✓ Individual level: age, gender, marital status, employment status, education, housing tenure, car access, ethnicity	✓ It does not evaluate quality or use of GS<br>✓ Only number of trees or private gardens evaluated<br>✓ Years that participants have been living in the area
White *et al.* 2013, The UK [27]	Longitudinal Adults (*N* = 12,818)	Fixed-effects regression	✓ CAU level: income, employment, education, crime<br>✓ Individual level: age, education, marital status, living with children, work-limiting health status, labourmarket status, residence type, household space, commute length	✓ It does not evaluate quality or use of GS<br>✓ Participants have been living at least 1 year in the studied residence<br>✓ Only urban areas included<br>✓ Gardens included
White *et al.* 2013, The UK [28]	Longitudinal Adults (*N* = 15,361)	Fixed-effects regression	✓ Individual level: age, education, marital status, living with children, work-limiting health status, labourmarket status, residence type, household space, commute length, green space	✓ It does not evaluate quality or use of blue spaces

CAU level: Census area unit level, GS: green space, BMI: body mass index.

Table S2. Criteria for quality assessment of the studies.

Study design	0 = ecological, 1 = cross-sectional, 2 = longitudinal
Confounding factors	0 = no confounding factors considered, 1 = confounding factors considered but some key confounders omitted, 2 = careful consideration of confounders
Statistics	0 = flaws in or inappropriate statistical testing or interpretation of statistical tests that may have affected results, 1 = appropriate statistical testing and interpretation of tests
Potential bias	0 = other study design or conduct issues that may have led to bias, 1 = no other serious study flaws
Multiplicity	0 = exposure of interest one of the many variables being tested, 1 = exposure of interest the main variable tested
Outcome assessment	0 = self-reported questionnaires, 1 = interviews conducted by experts or clinical records or other objective measures (biomarkers such as cortisol) that support the results of the mental health tests conducted
Green exposure assessment	0 = expert assessment (audit), 1 = satellite system or land-cover map
Use of green space	0 = not measured and/or not included in the analysis, 1=measured and included in the analysis
Quality of green space (as confounder)	0 = no, 1= yes, but partially, 2 = yes, and measured with an assessment tool
Effect size	0 = incomplete information, 1 = complete information (estimate and standard error or confidence interval).
Participants have been living at least 1 year in the studied area	0 = no or not clearly specified, 1= yes

Table S3. Specific scores for each item evaluated and the final quality scores and categories given to each study.

	Study Design	Confounding Factors	Statistics	Potential Bias	Multiplicity	Outcome Assessment	GS/BS Assessment	Use of GS/BS	Quality of GS/BS	Effect Size	At least 1y Living in the Area	Score (Absolute Number) [a]	Score (%) [a]	Quality Category
Alcock *et al.* 2014, The UK [1]	2	2	1	0	1	0	1	0	0	1	1	9	64	Good
Amoly 2014 et al., Spain [2]	1	2	1	0	0	0	1	1	0	1	0	7	50	Fair
Annerstedt *et al.* 2012, Sweden [3]	2	1	1	0	0	0	0	0	NA [a]	0	1	5	42	Fair
Araya *et al.* 2007, Chile [4]	1	2	1	0	1	1	0	0	0	1	0	7	50	Fair
Astell-Burt *et al.* 2013, Australia [5]	1	2	1	1	1	0	1	0	0	1	0	8	57	Fair
Astell-Burt *et al.* 2014, The UK [6]	2	2	1	1	1	0	1	0	0	1	1	10	71	Good
Balseviciene *et al.* 2014, Lithuania [7]	1	1	1	1	1	0	1	0	0	0	0	6	43	Fair
Beyer et al.2014, The USA [8]	1	2	1	0	1	0	1	0	0	1	0	7	50	Fair
De Vries *et al.* 2003, The Netherlands [9]	1	2	1	0	0	0	1	0	0	0	1	6	43	Fair
De Vries *et al.* 2013, The Netherlands [10]	1	2	0	0	1	0	0	0	NA [a]	1	0	5	42	Fair
Duncan *et al.* 2013, The USA [11]	1	1	0	1	0	0	1	0	0	1	0	5	36	Poor
Fan *et al.* 2011, The USA [12]	1	2	1	0	0	0	1	0	0	0	0	5	36	Poor

Table S3. *Cont.*

	Study Design	Confounding Factors	Statistics	Potential Bias	Multiplicity	Outcome Assessment	GS/BS Assessment	Use of GS/BS	Quality of GS/BS	Effect Size	At least 1y Living in the Area	Score (Absolute Number)[a]	Score (%)[a]	Quality Category
Flouri *et al.* 2014, The UK [13]	2	2	1	0	1	0	1	0	0	1	0	8	57	Fair
Francis *et al.* 2012, Australia [14]	1	2	1	1	0	0	1	0	2	0	1	9	64	Good
Maas *et al.* 2009, The Netherlands [15]	1	2	1	1	1	0	1	0	0	0	1	8	57	Fair
Maas *et al.* 2009, The Netherlands [16]	1	2	1	1	1	1	1	0	0	1	1	10	71	Good
Markevych *et al.* 2014, Germany [17]	1	2	1	0	1	0	1	0	0	1	1	8	57	Fair
Nutsford *et al.* 2013, New Zealand [18]	0	1	1	1	0	1	1	0	0	1	0	6	43	Fair
Reklaitiene *et al.* 2014, Lithuania [19]	1	1	1	1	1	0	1	1	0	1	0	8	57	Fair
Richardson *et al.* 2013, New Zealand [20]	1	1	1	1	1	0	1	0	0	1	0	7	50	Fair
Roe *et al.* 2013, The UK [21]	1	1	1	0	0	1	1	0	0	1	1	7	50	Fair
Sarkar *et al.* 2013, The UK [22]	1	2	1	1	0	0	1	0	1	0	0	7	50	Fair
Sturm *et al.* 2014, The USA [23]	1	1	1	0	1	0	0	1	0	1	0	6	43	Fair
Triguero-Mas *et al.* 2015, Spain [24]	1	2	1	1	0	0	1	0	0	1	0	7	50	Fair

Table S3. *Cont.*

	Study Design	Confounding Factors	Statistics	Potential Bias	Multiplicity	Outcome Assessment	GS/BS Assessment	Use of GS/BS	Quality of GS/BS	Effect Size	At least 1y Living in the Area	Score (Absolute Number)[a]	Score (%)[a]	Quality Category
Van den Berg *et al.* 2010, The Netherlands [25]	1	1	1	1	1	0	1	0	0	0	1	7	50	Fair
Weich *et al.* 2002, The UK [26]	1	1	1	1	0	0	0	0	0	1	1	6	43	Fair
White *et al.* 2013, The UK [27]	2	2	1	1	0	0	1	0	0	1	1	9	64	Good
White *et al.* 2013, The UK [28]	2	2	1	1	0	0	1	0	0	1	0	8	57	Fair

GS/BS: green space or blue space (depending on the studied exposure in each study); [a]For each study the total score was calculated by adding the scores on the 11 dimensions and expressing them as a percentage of the maximum score, which was 14, except for two studies [11,12] in which the inclusion of quality of green spaces as a confounder did not make sense as the main exposure of interest was the quality of green spaces (maximum score = 12). Afterwards, five categories were created to define the quality of each study: *excellent quality* (score ≥81%), *good quality* (between 61 and 80%), *fair quality* (between 41 and 60%), *poor quality* (between 21 and 40%) and *very poor quality* (≤20%).

References

1. Alcock, I.; White, M.P.; Wheeler, B.W.; Fleming, L.E.; Depledge, M.H. Longitudinal effects on mental health of moving to greener and less green urban areas. *Environ. Sci. Technol.* **2014**, *48*, 1247–1255.
2. Amoly, E.; Dadvand, P.; Forns, J.; López-Vicente, M.; Basagaña, X.; Julvez, J.; Alvarez-Pedrerol, M.; Nieuwenhuijsen, M.J.; Sunyer, J. Green and Blue Spaces and Behavioral Development in Barcelona Schoolchildren: The BREATHE Project. *Environ. Health Perspect.* **2014**, doi:10.1289/ehp.1408215.
3. Annerstedt, M.; Ostergren, P.-O.; Björk, J.; Grahn, P.; Skärbäck, E.; Währborg, P. Green qualities in the neighbourhood and mental health-results from a longitudinal cohort study in Southern Sweden. *BMC Public Health* **2012**, *12*, doi: 10.1186/1471-2458-12-337.
4. Araya, R.; Montgomery, A.; Rojas, G.; Fritsch, R.; Solis, J.; Signorelli, A.; Lewis, G. Common mental disorders and the built environment in Santiago, Chile. *Br. J. Psychiatr.* **2007**, *190*, 394–401.
5. Astell-Burt, T.; Feng, X.; Kolt, G.S. Mental health benefits of neighbourhood green space are stronger among physically active adults in middle-to-older age: Evidence from 260,061 Australians. *Prev. Med. (Baltim.)* **2013**, *57*, 601–606.
6. Astell-Burt, T.; Mitchell, R.; Hartig, T. The association between green space and mental health varies across the lifecourse. A longitudinal study. *J. Epidemiol. Commun. Health* **2014**, *68*, 578–583.
7. Balseviciene, B.; Sinkariova, L.; Grazuleviciene, R.; Andrusaityte, S.; Uzdanaviciute, I.; Dedele, A.; Nieuwenhuijsen, M.J. Impact of residential greenness on preschool children's emotional and behavioral problems. *Int. J. Environ. Res. Public Health* **2014**, *11*, 6757–6770.
8. Beyer, K.M. M.; Kaltenbach, A.; Szabo, A.; Bogar, S.; Nieto, F.J.; Malecki, K.M. Exposure to neighborhood green space and mental health: Evidence from the survey of the health of Wisconsin. *Int. J. Environ. Res. Public Health* **2014**, *11*, 3453–3472.
9. De Vries, S.; Verheij, R.A.; Groenewegen, P.P.; Spreeuwenberg, P. Natural environments–Healthy environments? An exploratory analysis of the relationship between greenspace and health. *Environ. Plan. A* **2003**, *35*, 1717–1731
10. De Vries, S.; van Dillen, S.M.E.; Groenewegen, P.P.; Spreeuwenberg, P. Streetscape greenery and health: Stress, social cohesion and physical activity as mediators. *Soc. Sci. Med.* **2013**, *94*, 26–33.
11. Duncan, D.T.; Piras, G.; Dunn, E.C.; Johnson, R.M.; Melly, S.J.; Molnar, B.E. The built environment and depressive symptoms among urban youth: A spatial regression study. *Spat. Spatiotemporal. Epidemiol.* **2013**, *5*, 11–25.
12. Fan, Y.; Das, K. V; Chen, Q. Neighborhood green, social support, physical activity, and stress: Assessing the cumulative impact. *Health Place* **2011**, *17*, 1202–1211.
13. Flouri, E.; Midouhas, E.; Joshi, H. The role of urban neighbourhood green space in children's emotional and behavioural resilience. *J. Environ. Psychol.* **2014**, *40*, 179–186.
14. Francis, J.; Wood, L.J.; Knuiman, M.; Giles-Corti, B. Quality or quantity? Exploring the relationship between Public Open Space attributes and mental health in Perth, Western Australia. *Soc. Sci. Med.* **2012**, *74*, 1570–1577.

15. Maas, J.; van Dillen, S.M. E.; Verheij, R.A.; Groenewegen, P.P. Social contacts as a possible mechanism behind the relation between green space and health. *Health Place* **2009**, *15*, 586–595.
16. Maas, J.; Verheij, R.A.; de Vries, S.; Spreeuwenberg, P.; Schellevis, F.G.; Groenewegen, P.P. Morbidity is related to a green living environment. *J. Epidemiol. Commun. Health* **2009**, *63*, 967–973.
17. Markevych, I.; Tiesler, C.M. T.; Fuertes, E.; Romanos, M.; Dadvand, P.; Nieuwenhuijsen, M.J.; Berdel, D.; Koletzko, S.; Heinrich, J. Access to urban green spaces and behavioural problems in children: Results from the GINIplus and LISAplus studies. *Environ. Int.* **2014**, *71*, 29–35.
18. Nutsford, D.; Pearson, A.L.; Kingham, S. An ecological study investigating the association between access to urban green space and mental health. *Public Health* **2013**, *127*, 1005–1011.
19. Reklaitiene, R.; Grazuleviciene, R.; Dedele, A.; Virviciute, D.; Vensloviene, J.; Tamosiunas, A.; Baceviciene, M.; Luksiene, D.; Sapranaviciute-Zabazlajeva, L.; Radisauskas, R.; *et al.* The relationship of green space, depressive symptoms and perceived general health in urban population. *Scand. J. Public Health* **2014**, doi:10.1177/1403494814544494.
20. Richardson, E.A.; Pearce, J.; Mitchell, R.; Kingham, S. Role of physical activity in the relationship between urban green space and health. *Public Health* **2013**, *127*, 318–324.
21. Roe, J.J.; Thompson, C.W.; Aspinall, P.A.; Brewer, M.J.; Duff, E.I.; Miller, D.; Mitchell, R.; Clow, A. Green space and stress: Evidence from cortisol measures in deprived urban communities. *Int. J. Environ. Res. Public Health* **2013**, *10*, 4086–4103.
22. Sarkar, C.; Gallacher, J.; Webster, C. Urban built environment configuration and psychological distress in older men: Results from the Caerphilly study. *BMC Public Health* **2013**, *13*, doi:10.1186/1471-2458-13-695.
23. Sturm, R.; Cohen, D. Proximity to urban parks and mental health. *J. Ment. Health Policy Econ.* **2014**, *17*, 19–24.
24. Triguero-Mas, M.; Dadvand, P.; Cirach, M.; Martínez, D.; Medina, A.; Mompart, A.; Basagaña, X.; Gražulevičienė, R.; Nieuwenhuijsen, M.J. Natural outdoor environments and mental and physical health: Relationships and mechanisms. *Environ. Int.* **2015**, *77*, 35–41.
25. Van den Berg, A.E.; Maas, J.; Verheij, R.A.; Groenewegen, P.P. Green space as a buffer between stressful life events and health. *Soc. Sci. Med.* **2010**, *70*, 1203–1210.
26. Weich, S.; Blanchard, M.; Prince, M.; Burton, E.; Erens, B.; Sproston, K. Mental health and the built environment: Cross-sectional survey of individual and contextual risk factors for depression. *Br. J. Psychiatry* **2002**, *180*, 428–433.
27. White, M.P.; Alcock, I.; Wheeler, B.W.; Depledge, M.H. Would you be happier living in a greener urban area? A fixed-effects analysis of panel data. *Psychol. Sci.* **2013**, *24*, 920–928.
28. White, M.P.; Alcock, I.; Wheeler, B.W.; Depledge, M.H. Coastal proximity, health and well-being: Results from a longitudinal panel survey. *Health Place* **2013**, *23*, 97–103.

© 2015 by the authors; licensee MDPI, Basel, Switzerland. This article is an open access article distributed under the terms and conditions of the Creative Commons Attribution license (http://creativecommons.org/licenses/by/4.0/).
